# Examining Associations between Self-Rated Health and Proficiency in Literacy and Numeracy among Immigrants and U.S.-Born Adults: Evidence from the Program for the International Assessment of Adult Competencies (PIAAC)

**DOI:** 10.1371/journal.pone.0130257

**Published:** 2015-07-01

**Authors:** Esther Prins, Shannon Monnat

**Affiliations:** 1 Adult Education Program, Goodling Institute for Research in Family Literacy, and Institute for the Study of Adult Literacy, The Pennsylvania State University, University Park, Pennsylvania, United States of America; 2 Department of Agricultural Economics, Sociology, and Education, and Population Research Institute, The Pennsylvania State University, University Park, Pennsylvania, United States of America; UNC School of Dentistry, University of North Carolina-Chapel Hill, UNITED STATES

## Abstract

This paper uses data from the Program for the International Assessment of Adult Competencies (PIAAC) to analyze the relationship between self-reported health (SRH) and literacy and numeracy proficiency for immigrants compared to U.S.-born respondents and for Hispanic versus Asian immigrants. The research questions were: (1) Are literacy and numeracy scores associated with adults’ SRH? (2) Are associations between SRH and literacy and numeracy proficiency moderated by immigrant status? (3) Among immigrants, are literacy and numeracy scores more strongly associated with SRH for Hispanics versus Asians? Immigrants had significantly lower literacy and numeracy scores, yet reported better health than U.S.-born respondents. Ordinal logistic regression analyses showed that literacy and numeracy were both positively related to SRH for immigrants and U.S.-born adults, and should therefore be viewed as part of the growing evidence that literacy is an independent and significant social determinant of health. Second, U.S.-born and immigrant adults accrued similarly positive health benefits from stronger literacy and numeracy skills. Third, although Hispanic immigrants were more disadvantaged than Asian immigrants on almost all socioeconomic characteristics and had significantly lower literacy and numeracy scores and worse SRH than Asian immigrants, both Hispanic and Asian immigrants experienced similar positive health returns from literacy and numeracy proficiency. These findings underscore the potential health benefits of providing adult basic education instruction, particularly for immigrants with the least formal schooling and fewest socioeconomic resources.

## Introduction

This paper uses U.S. data from the Program for the International Assessment of Adult Competencies (PIAAC) to analyze the relationship between self-rated health (SRH) and proficiency in literacy and numeracy for immigrants and U.S.-born adults. Educational attainment is strongly related to health [1, 2]; however, the health benefits of formal education do not accrue equally across racial/ethnic groups in the United States. In particular, blacks experience “diminishing returns to education,” meaning they derive fewer health rewards than whites from increasing levels of formal education [3–5]. Our previous PIAAC analyses suggest that literacy and numeracy proficiency are also associated with U.S. adults’ self-rated health, but unlike with educational attainment, minority racial/ethnic groups benefit equally from stronger literacy and numeracy skills [6]. However, this previous research did not explore how the relationship between health and proficiency in literacy and numeracy varies by immigrant status, a particularly important topic given the large and growing immigrant population in the U.S. This is also an under-explored topic in the literature on basic skills and health.

Immigrants to the U.S. tend to enjoy better health than their U.S.-born counterparts. This “healthy immigrant effect” primarily stems from selection effects—healthier people are more likely to migrate—and have better health behaviors, at least immediately following migration [7–9]. However, we do not know whether immigrants and U.S.-born adults accumulate similar health benefits from literacy and numeracy. On the one hand, inequitable access to health care and insurance [10, 11], poor healthcare quality [10], segregation and environmental hazards [12], cultural dissonance and lack of familiarity with the healthcare system [13], and limited English proficiency [10] may diminish immigrants’ ability to convert literacy and numeracy proficiencies into health rewards. On the other hand, because people with more socioeconomic resources are more likely to migrate, immigrants may be better equipped to reap health benefits from literacy and numeracy.

Furthermore, Asian and Hispanic immigrants’ heterogeneous cultural, employment, and residential contexts might influence their ability to accumulate health rewards from basic skills. Specifically, Asian immigrants might be better able to do so because they have comparatively higher educational attainment and more highly educated parents and tend to work in professional occupations [14].

Our research explores these topics by answering the following questions: (1) Are literacy and numeracy scores associated with adults’ SRH? (2) Are associations between SRH and proficiency in literacy and numeracy moderated by immigrant status? (3) Among immigrants, are literacy and numeracy skills more strongly associated with SRH for Hispanics versus Asians? We also examine the role of socioeconomic status (SES)-related human capital characteristics as a pathway through which literacy and numeracy may be associated with SRH. This study adds to scholarship on social determinants of health and adult basic skills [15–19] by examining how literacy and numeracy may differentially contribute to health for immigrants and native-born adults in the United States.

## Literacy, Numeracy, and Health among Immigrants

The literature on the social determinants of health posits that economic and social opportunities and resources such as educational attainment are a fundamental cause of health and health disparities [20–24]. Accordingly, we view literacy and numeracy proficiency as tools that adults can use to access the economic and social opportunities and resources needed to maintain and improve health. People who struggle with basic skills are also excluded from the resources and opportunities (e.g., employment, income, education) that enable people to flourish.

Fifty percent of PIAAC respondents scored in the bottom two literacy proficiency levels, compared to 60% for numeracy [25]. (PIAAC literacy and numeracy scores range from 0 to 500, and correspond to five proficiency levels [Below Level 1 to Level 4/5]. See the National Center for Education Statistics for more information on proficiency levels: http://nces.ed.gov/surveys/piaac/measure.asp.) The average literacy and math scores for U.S. adults are 270 and 253, respectively, which corresponds to Level 2. At Level 2 or below, adults are more likely to have difficulty understanding denser, more complicated types of print and performing more complex numeracy-related tasks. Immigrants—particularly Hispanics—had significantly lower numeracy and literacy scores than U.S.-born respondents [26], and “both immigrants and natives with low literacy scores were more likely to report poor health” [27]. These results mirror the findings on immigrants, health literacy, and health status in the 2003 National Assessment of Adult Literacy [28–30].

Due to social stratification, the likelihood of struggling with literacy, numeracy, and poor health chiefly affects people of color [28, 31–33], the elderly [28, 33], and adults with limited income [28, 32–34], formal education [28, 31, 33, 34], and English proficiency [28], characteristics that we control for in our analyses.

People’s literate capabilities may influence their health in myriad ways, including their ability to read, analyze, and use health-related texts. Overall, adults with unmet literacy needs tend to have worse health, “including knowledge, intermediate disease markers, measures of morbidity, general health status, and use of health resources” [34, 35(1228), 36–41]. These patterns extend to immigrants, especially Hispanics [42], the elderly [43], and non-native English speakers [44].

We found only one study examining the relationship between *print* literacy (rather than *health* literacy) proficiency and health status among immigrants [45]. For every racial/ethnic group except Blacks, U.S.-born respondents had significantly higher literacy scores on the 1992 National Adult Literacy Survey than their foreign-born peers [46], yet U.S.-born adults had much greater odds of having a long-term illness or a condition that kept them from work [45]. These findings illustrate the immigrant health paradox of having better health despite fewer socioeconomic resources—and lower literacy scores—than U.S.-born peers [9].

Compared to the research on literacy, we know much less about numeracy and health among immigrants. Mathematical calculations, reasoning, and understanding influence risk assessment and decision making (e.g., estimating the probability of developing cancer) [47, 48], interpretation of numerical and graphical information [32], and health behaviors such as managing medications [49–51].

Prior analyses of immigrants’ numeracy skills on health-related tasks (using quantitative items from the Test of Functional Health Literacy in Adults) reveal mixed results. Some immigrant groups (e.g., Korean immigrant women) had high numeracy scores [88], whereas others, such as Spanish-speaking adults, had low scores and struggled with tasks such as medication dosing [52].

Compared to the research on literacy, however, the empirical evidence on numeracy and health outcomes is sparser and less conclusive [37, 53]. In addition, our prior PIAAC study [6] showed that after adjusting for background characteristics, numeracy was not significantly related to U.S. adults’ health. We found no studies analyzing the relationship between numeracy and immigrants’ SRH or whether this differs for native- versus foreign-born adults. Thus, the current paper tests whether our previous findings apply to native- and foreign-born adults.

## Methods

### Data Source

Data are from the public use files of the 2012 Program for the International Assessment of Adult Competencies (PIAAC), an international survey of adults (16–65) in 24 countries that measures literacy, numeracy, and technological problem solving. We analyzed only the United States PIAAC assessment due to the country’s unique history and context of immigration. A total of 5,010 U.S. respondents completed the survey. The background questionnaire was available in English and Spanish, whereas the literacy and numeracy assessments were conducted only in English.

### Variables

The outcome of interest was self-rated health (SRH). Respondents were asked: “In general, would you say your health is excellent, very good, good, fair, or poor?” SRH is a comprehensive, accurate measure of health outcomes in the U.S. and internationally [54]. We maintained SRH in its 5-level ordinal scale. Literacy and numeracy scores were the main predictor variables. In the PIAAC data, each respondent was assigned ten plausible value scores for both literacy and numeracy skills. Respondents were not administered every literacy and numeracy question; instead, they responded to a fraction of the literacy and numeracy assessments. Thus, plausible values were developed as computational approximations to obtain consistent estimates of literacy and numeracy. These are imputed values that resemble individual test scores and have approximately the same distribution as actual values. We employed the special analytic techniques that were designed for use with these plausible values [55, 56].

Our moderator of interest was U.S.-born versus foreign-born (immigrant) status. For the second part of our analyses, we also examined differences in associations between SRH and proficiency in literacy and numeracy between immigrant Hispanics and immigrant Asians. We focused on these two groups because the sample sizes for other immigrant groups (e.g., Black immigrants) are too small for robust regression analyses. We controlled for several variables that have been found to influence SRH in previous research [54]: age (24 or less [ref], 25–34, 35–44, 45–54, 55 or older); sex (male = ref); race/ethnicity (non-Hispanic white [ref], non-Hispanic black, Hispanic, Asian/Pacific Islander, other race); household size; whether respondent lives with a spouse/partner; any children aged 12 or younger; U.S. census region (West [ref], Northeast, Midwest, and South); any vision or hearing problem or diagnosed learning disability; has health insurance; and received a flu shot in the past year as a measure of health care utilization. We excluded the other health care utilization measures because they are sex- and/or age-specific (e.g., mammogram).

We hypothesized that differential accrual of the human capital resources that are often viewed as measures of assimilation may be a pathway through which immigrant status could condition or moderate associations between proficiency in literacy and numeracy and SRH. These human capital characteristics included: (1) educational attainment (did not complete high school [ref], high school graduate/some college, certificate from trade school or other, associate degree, bachelor’s degree, and master’s degree or higher); (2) employment status (employed [ref], unemployed, pupil/student/apprentice/internship, retired, unable to work due to disability, and homemaker or other); (3) mother’s and father’s educational attainment (did not complete high school [ref], completed high school, attended college or more); (4) an English proficiency score comprised of a summed measure of respondents’ self-reported ability to speak, read, write, and understand spoken English (higher scores represent greater proficiency); and (5) income quintile (fifth quintile = ref).

Because the income question asks only about employment income and excludes income from transfers (e.g., retirement, social security, public assistance) and property (e.g., rents), 35% of the responses are missing. These respondents are more likely than those with a valid response to be in the youngest or oldest age categories, to have not completed high school, and to be unemployed. To include these respondents in our analyses and still be able to control for income, we created a “missing income” category for people with no reported income, and we included them with our income variable. After deletion of cases with missing information on our other variables, our analytic sample size was 4,646.

For analyses of immigrant Asians and Hispanics (N = 420), we also controlled for years in the U.S. (5 or fewer [ref], 6–10, 11–15, and more than 15) and age when respondent learned English (learned English as a first language [ref], learned English before age 16, or learned English at 16 years or older). A review of multicollinearity diagnostics revealed no concerns when including these variables in the same models.

### Statistical Analyses

Descriptive statistics were calculated by U.S.-born versus foreign-born (immigrant) as means and percentages as appropriate, and we used two-tailed difference of means/proportions t-tests to identify whether there were significant differences in sample characteristics across the two groups of respondents. These analyses were repeated separately for Hispanic and Asian immigrants. To examine bivariate associations between SRH and proficiency in literacy and numeracy, we plotted mean literacy and numeracy scores across categories of SRH separately for U.S.-born and immigrant respondents and also separately for Hispanic and Asian immigrants. We present error bars representing 95% confidence intervals (CI) for all categories.

Ordinal logistic regression with Stata MP 13 PIAACREG proceeded as follows. First, to establish a baseline, we assessed the association between literacy and SRH and numeracy and SRH for the whole sample and report the unadjusted odds ratio (OR) along with 95% CI and p-value. Literacy and numeracy were strongly correlated, so we could not include them in the same regression models. Second, we adjusted those models for the demographic and health characteristics listed above. Third, we adjusted for the human capital resources listed above. To assess our main research questions, we then repeated those analyses separately by U.S.-born versus immigrant status to determine whether (a) literacy and/or numeracy were associated with SRH for one group but not the other and (b) whether human capital characteristics drove any differences between the two groups. Within the whole sample, we then tested for statistically significant interactions between immigrant status and (a) literacy and (b) numeracy. Finally, we restricted our sample to immigrant Asians and Hispanics and used the same regression models to determine whether literacy/numeracy proficiency is more strongly associated with SRH for Asian versus Hispanic immigrants.

## Results

### Differences in Sample Characteristics between U.S.-Born and Immigrant Respondents

There were significant differences between the characteristics of U.S.-born (N = 4,033) versus immigrant respondents (N = 613), as shown in Table 1. U.S.-born respondents had higher average literacy and numeracy scores than immigrants (p<.001). Though there were no significant differences in the percentages of U.S.-born versus immigrants who reported excellent, good, fair or poor health, a significantly greater percentage (p = .013) of U.S.-born respondents (34.5%) reported very good health compared to immigrant respondents (29.7%). U.S.-born adults were more likely than immigrants to have some sort of disability (p = .010) and to have health insurance (p<.001).

**Table 1:** Descriptive Statistics for Sample Characteristics by U.S.-Born versus Immigrant.

	U.S.-Born	Immigrant	t-value	p
Percentages or mean (standard deviation)	(N = 4,033)	(N = 613)		
Literacy Score^a^	277.03 (44.96)	240.59 (56.52)	15.25	<.001
Numeracy Score^b^	260.13 (52.51)	227.77 (66.16)	11.57	<.001
*Self-Rated Health*				
Excellent	23.9	25.7	-1.04	0.33
Very Good	34.5	29.7	2.49	0.01
Good	27.8	28.8	-0.52	0.60
Fair	10.3	13.0	-1.84	0.07
Poor	3.5	2.9	0.90	0.37
*Demographic Characteristics*				
Age				
24 or less	19.4	10.6	6.27	<.001
25–34	19.9	24.7	-2.55	<.001
35–44	19.0	27.1	-4.26	<.001
45–54	21.6	22.8	-0.67	0.50
55 or older	20.2	14.8	3.27	0.001
Female	51.0	52.1	-0.51	0.61
Race/Ethnicity				
Non-Hispanic white	75.2	18.2	32.55	<.001
Non-Hispanic black	11.8	9.5	1.75	0.08
Hispanic	8.4	46.5	-18.46	<.001
Asian/Pacific Islander	2.0	24.4	-12.84	<.001
Other race	2.6	1.4	2.30	0.02
Number of people living in household	3.12 (1.47)	3.78 (1.80)	-9.17	<.001
Lives with a spouse or partner	58.7	68.3	-4.70	<.001
Has children aged 12 or younger	20.4	32.4	-6.01	<.001
Region				
Northeast	17.5	22.5	-2.82	<.001
Midwest	23.8	9.8	10.21	<.001
South	37.6	35.3	1.14	0.26
West	21.2	32.4	-5.63	<.001
*Health Background*				
Has vision/hearing problems or diagnosed learning disability	23.4	19.0	2.58	0.01
Has health insurance	82.4	65.1	8.56	<.001
Received flu shot in past year	38.5	41.5	-1.41	0.16
*Human Capital*				
Educational Attainment				
Did not complete high school	11.3	26.2	-8.08	<.001
High school graduate/some college	42.3	32.5	4.87	<.001
Certificate from trade school or other	9.4	4.6	4.97	<.001
Associate degree	9.8	5.4	4.25	<.001
Bachelor’s degree	17.3	15.0	1.47	0.14
Master’s degree or higher	10.0	16.3	-4.05	<.001
Employment Status				
Employed	64.5	68.6	-2.09	0.04
Unemployed	7.8	8.0	-0.20	0.84
Pupil, student, apprentice, internship	10.6	7.4	2.67	0.01
Retired	3.7	2.2	2.28	0.02
Disabled	4.9	2.2	3.96	<.001
Homemaker or other	8.5	11.5	-2.20	0.03
Income				
First quintile (lowest)	13.6	15.4	-1.15	0.25
Second quintile	12.7	18.1	-3.27	0.001
Third quintile	13.9	12.0	1.37	0.17
Fourth quintile	13.7	10.0	2.75	0.01
Fifth quintile (highest)	14.0	12.5	1.08	0.28
Income not reported	32.1	32.0	0.02	0.99
English proficiency level (Range 0–12; higher score = better)	11.62 (1.10)	8.3 (3.99)	21.96	<.001
Mother’s Educational Attainment				
Did not complete high school	20.9	53.8	-15.55	<.001
Completed high school	51.3	24.9	13.73	<.001
Attended college or more	27.8	21.2	3.60	<.001
Father’s Educational Attainment				
Did not complete high school	23.7	46.5	-1.73	<.001
Completed high school	48.0	26.6	10.59	<.001
Attended college or more	28.3	27.0	0.70	0.49

^a^ U.S.-born respondents’ mean literacy score (277) is just above the Level 3 threshold (276–325), whereas immigrants’ mean score (241) corresponds to lower Level 2 (226–275).

^b^ U.S.-born and immigrant respondents’ mean numeracy scores both correspond to Level 2 (226–275), but they fall at the higher (260) and lower (228) ends of Level 2, respectively.

*Note*: Means and standard deviations reported for continuous variables. Percentages reported for categorical variables; Difference of means/percentage t-tests to determine sig of differences, p-values represent two-tailed tests

N = 4,646; weighted values

U.S.-born adults were more likely than immigrants to be in the youngest (24 or less) and oldest (55 and older) age categories (p = .001). U.S.-born respondents were significantly more likely to be non-Hispanic white (p<.001) and significantly less likely to be Hispanic or Asian (p<.001). Immigrants had a higher average household size (p<.001) and were more likely to be living with a spouse or partner (p<.001) and to have children under 12 (p<.001) than U.S.-born adults. Immigrants were more likely than U.S.-born respondents to live in the Northeast (p<.001) or West (p<.001), whereas U.S.-born respondents were significantly more likely than immigrants to live in the Midwest (p<.001).

Regarding human capital characteristics, immigrants were more likely than the U.S.-born both to not complete high school (p<.001) and to have a master’s degree or higher (p<.001), suggesting that this sample represents a divergent population of immigrants. Immigrants were also more likely than U.S.-born adults to be employed (p = .037) or a homemaker (p = .028), and less likely to be a student (p = .008), retired (p = .023), or unable to work due to a disability (p<.001). There were no major differences in representation in the highest and lowest income percentiles, but immigrants were more likely to be in the second quintile (p<.001) whereas U.S.-born adults were more likely to be in the fourth quintile (p = .006), suggesting higher income. There were no significant differences in the percentage of each group that reported no income (p = 0.987). U.S.-born respondents had significantly better English proficiency (p<.001), and their parents had higher average educational attainment.

**Fig 1:**
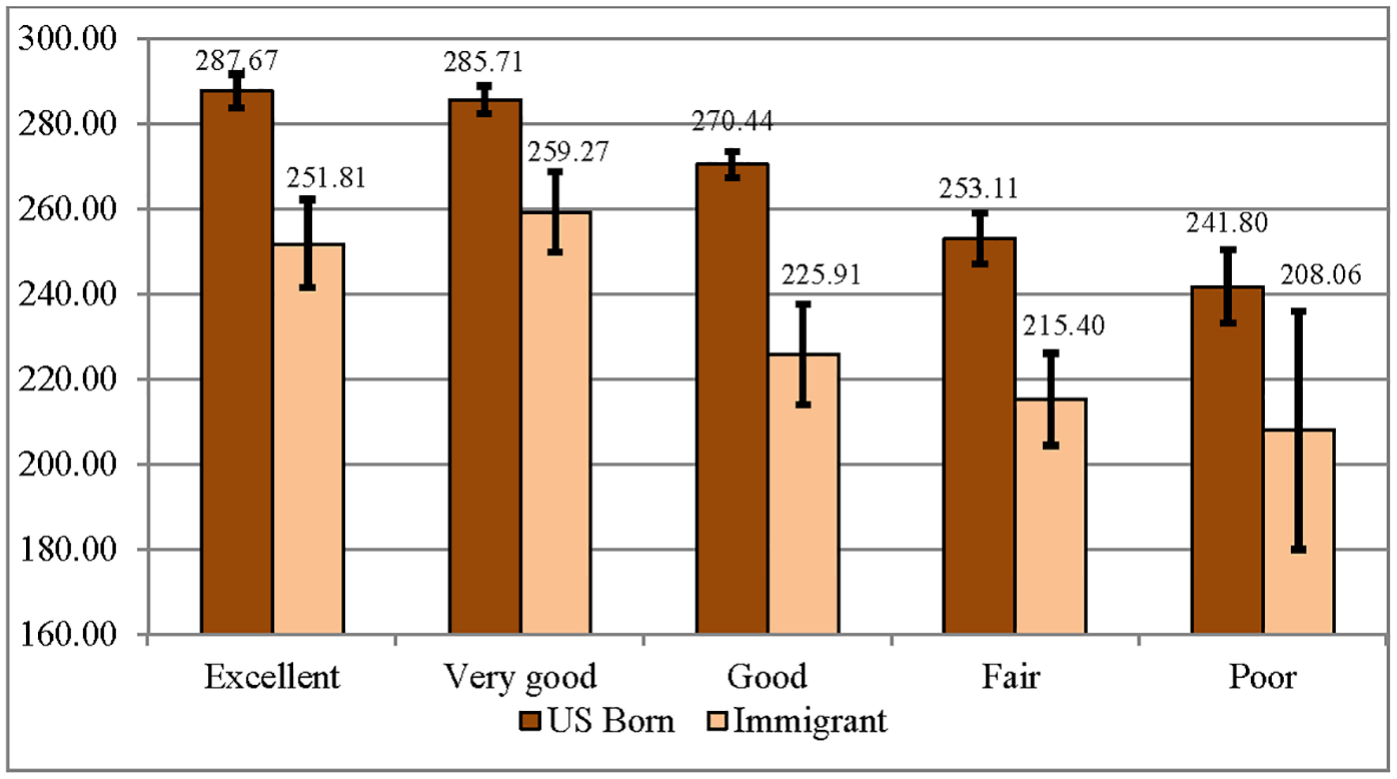
Average Literacy Scores by Self-Rated Health Category for U.S.-Born and Immigrant Respondents (95% CIs) (N = 4,664).

**Fig 2:**
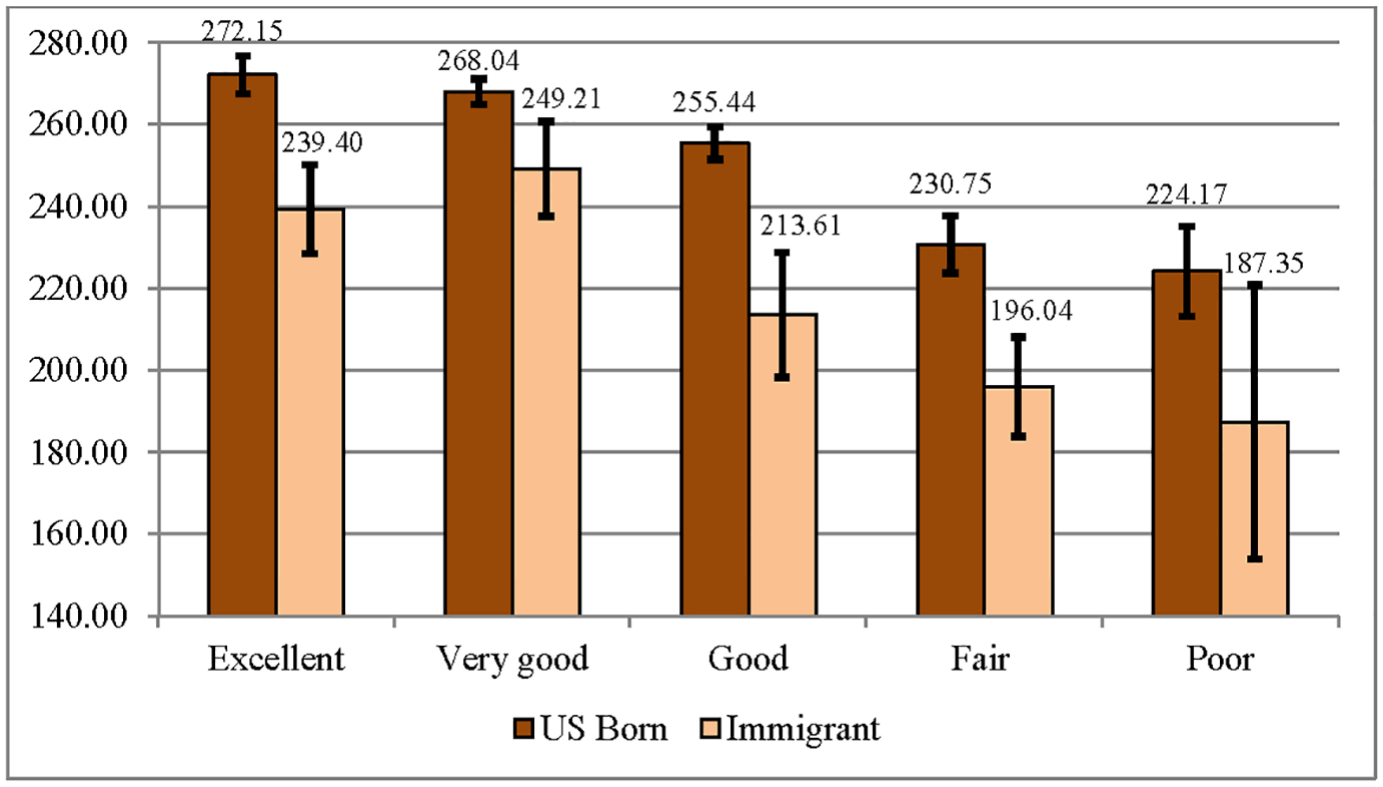
Average Numeracy Scores by Self-Rated Health Category for U.S.-Born and Immigrant Respondents (95% CIs) (N = 4,664).

Bivariate associations between literacy and SRH for U.S.-born and immigrant adult respondents demonstrated average positive associations for both groups (Figs 1 and 2). Among the U.S.-born, average literacy and numeracy scores were significantly higher among those who report excellent, very good, or good health compared to those who report fair or poor health. Among immigrants, average literacy and numeracy scores were significantly higher among those who report excellent and very good health compared to those who report good, fair, or poor health.

### Associations between Proficiency in Literacy and Numeracy Proficiency and Self-Rated Health

Unadjusted ordinal logistic regression models demonstrated positive associations between literacy and SRH (UOR = 1.11, 95% CI = 1.09–1.12, p<.001) and numeracy and SRH (UOR = 1.09, 95% CI = 1.07–1.10, p<.001). For meaningful interpretation, we scaled the literacy and numeracy values to 10-unit increments. Therefore, a 10-point increase in literacy was associated with about 11% greater odds of being in a better SRH category, and a 10-point increase in numeracy was associated with about 9% greater odds of being in a better SRH category.

Adjusting the regression models for demographic and health background characteristics in Models 1a and 1b (Table 2) did little to change the associations between literacy and numeracy with SHR. However, the addition of human capital characteristics in Models 2a and 2b (Table 2) decreased the odds ratios for both literacy and numeracy. There was no longer a significant association between numeracy and SHR (AOR = 1.01, 95% CI = 0.99–1.03, p = 0.32). Further, the association for literacy was reduced from a 11% to a 3% increase in odds of being in a better health category (AOR = 1.03, 95% CI = 1.00–1.05, p = 0.02). These findings suggest that human capital resources may serve as a pathway through which literacy and numeracy proficiency is related to SRH. Notably, our results on U.S.-born versus foreign-born differences in SRH illustrated the immigrant health paradox; after controlling for background characteristics in both the literacy and numeracy models, immigrants had better SRH.

**Table 2:** Adjusted Odds Ratios and 95% Confidence Intervals from Ordinal Logistic Regressions of Self-Rated Health on Literacy and Numeracy.

	Literacy				Numeracy			
	Model 1a		Model 2a		Model 1b		Model 2b	
	AOR	p	AOR	p	AOR	p	AOR	p
Literacy	1.11 (1.09–1.12)	< .001	1.03 (1.00–1.05)	0.02	-----	-----	-----	
Numeracy	-----		-----		1.07 (1.05–1.08)	< .001	1.01 (0.99–1.03)	0.32
*Demographic Characteristics*								
Immigrant Status								
U.S.-Born (ref)	1		1		1		1	
Foreign-Born	1.46 (1.20–1.77)	< .001	1.49 (1.17–1.92)	0.002	1.36 (1.12–1.64)	0.002	1.47 (1.14–1.89)	0.00
Age								
24 or less (ref)	1		1		1		1	
25–34	0.66 (0.54–0.81)	< .001	0.60 (.046–0.79)	< .001	0.65 (0.54–0.79)	< .001	0.60 (0.46–0.79)	< .001
35–44	0.49 (0.38–0.62)	< .001	0.46 (0.35–0.62)	< .001	0.48 (0.38–0.61)	< .001	0.46 (0.35–0.62)	< .001
45–54	0.43 (0.34–0.56)	< .001	0.43 (0.32–0.60)	< .001	0.42 (0.33–0.54)	< .001	0.43 (0.31–0.59)	< .001
55 or older	0.35 (0.28–0.43)	< .001	0.45 (0.39–0.50)	< .001	0.33 (0.27–0.41)	< .001	0.43 (0.33–0.57)	< .001
Female	0.90 (0.79–1.02)	0.07	0.45 (0.39–0.50)	< .001	0.98 (0.87–1.11)	0.78	0.93 (0.83–1.05)	0.27
Race/Ethnicity								
Non-Hispanic white (ref)	1		1		1		1	
Non-Hispanic black	0.90 (0.75–1.08)	0.24	0.86 (0.71–1.05)	0.14	0.94 (0.79–1.13)	0.52	0.84 (0.69–1.02)	0.08
Hispanic	0.85 (0.67–1.07)	0.17	1.04 (0.81–1.33)	0.77	0.82 (0.65–1.03)	0.09	1.02 (0.79–1.30)	0.91
Asian/Pacific Islander	0.80 (0.63–1.02)	0.07	0.68 (0.52–0.89)	0.005	0.76 (0.60–0.97)	0.03	0.67 (0.51–0.87)	0.003
Other race	0.83 (0.55–1.27)	0.39	0.95 (0.65–1.39)	0.78	0.85 (0.56–1.29)	0.45	0.95 (0.64–1.39)	0.78
Number of people living in household	0.99 (0.94–1.04)	0.62	1.02 (0.97–1.08)	0.42	0.99 (0.94–1.04)	0.66	1.02 (0.97–1.08)	0.42
Lives with a spouse or partner	1.28 (1.14–1.44)	< .001	1.10 (0.99–1.23)	0.08	1.25 (1.12–1.40)	< .001	1.10 (0.99–1.23)	0.08
Has children aged 12 or younger	1.08 (0.90–1.31)	0.40	1.05 (0.86–1.28)	0.62	1.08 (0.89–1.30)	0.46	1.04 (0.86–1.27)	0.66
Region								
West (ref)	1		1		1		1	
Northeast	1.24 (0.94–1.63)	0.13	1.19 (0.95–1.48)	0.14	1.24 (0.94–1.62)	0.13	1.17 (0.91–1.52)	0.23
Midwest	0.99 (0.75–1.30)	0.92	1.00 (0.80–1.25)	0.99	0.98 (0.75–1.29)	0.90	0.99 (0.79–1.24)	0.94
South	0.91 (0.72–1.15)	0.43	0.95 (0.78–1.16)	0.63	0.90 (0.72–1.14)	0.40	0.95 (0.78–1.15)	0.59
*Health Background*								
Has vision/hearing problems or diagnosed learning disability	0.46 (0.40–0.54)	< .001	1.09 (0.80–1.47)	0.59	0.46 (0.39–0.54)	< .001	0.59 (0.50–0.70)	< .001
Has health insurance	1.26 (1.07–1.48)	0.005	1.17 (0.98–1.39)	0.08	1.28 (1.09–1.50)	0.002	1.18 (0.99–1.41)	0.06
Received flu shot in past year	1.05 (0.91–1.22)	0.48	1.02 (0.88–1.18)	0.81	1.07 (0.92–1.23)	0.37	1.02 (0.88–1.18)	0.82
*Human Capital*								
Educational Attainment								
Did not complete high school (ref)			1				1	
High school graduate/some college			1.07 (0.86–1.34)	0.55			1.09 (0.84–1.41)	0.51
Certificate from trade school or other			1.09 (0.76–1.58)	0.63			1.12 (0.77–1.61)	0.55
Associate degree			1.27 (0.95–1.70)	0.10			1.32 (0.99–1.76)	0.06
Bachelor’s degree			1.82 (1.33–2.50)	< .001			1.91 (1.40–2.61)	< .001
Master’s degree or higher			2.04 (1.47–2.82)	< .001			2.17 (1.58–3.00)	< .001
Employment Status								
Employed (ref)			1				1	
Unemployed			0.71 (0.50–1.03)	0.07			0.72 (0.50–1.03)	0.07
Pupil, student, apprentice, internship			1.04 (0.75–1.45)	0.81			1.06 (0.76–1.48)	0.72
Retired			0.72 (0.51–1.02)	0.07			0.73 (0.51–1.03)	0.07
Disabled			0.05 (0.03–0.07)	< .001			0.05 (0.03–0.07)	< .001
Homemaker or other			0.94 (0.71–1.25)	0.68			0.95 (0.72–1.26)	0.72
Income								
First quintile			0.72 (0.56–0.94)	0.01			0.71 (0.55–0.92)	0.01
Second quintile			0.87 (0.67–1.14)	0.31			0.85 (0.65–1.11)	0.22
Third quintile			0.91 (0.72–1.15)	0.44			0.89 (0.71–1.12)	0.32
Fourth quintile			0.98 (0.78–1.22)	0.84			0.97 (0.77–1.21)	0.77
Fifth quintile (ref)			1				1	
Income not reported			0.76 (0.63–0.93)	0.01			0.75 (0.62–0.91)	0.004
English proficiency level (higher score = better)			1.08 (1.04–1.13)	< .001			1.09 (1.05–1.14)	< .001
Mother’s Educational Attainment								
Did not complete high school (ref)			1				1	
Completed high school			1.24 (1.05–1.46)	0.01			1.25 (1.06–1.47)	0.01
Attended college or more			1.19 (0.97–1.48)	0.10			1.22 (0.99–1.51)	0.06
Father’s Educational Attainment								
Did not complete high school (ref)			1				1	
Completed high school			1.05 (0.91–1.20)	0.51			1.05 (0.92–1.21)	0.46
Attended college or more			1.36 (1.13–1.64)	0.001			1.38 (1.14–1.67)	< .001

N = 4,646

Two-tailed tests

AOR = Adjusted Odds Ratio

### Immigrant Status, Literacy and Numeracy, and Self-Rated Health

We next examined associations between literacy and numeracy proficiency and SRH separately for U.S.-born (N = 4,033) and immigrant respondents (N = 613) by running unadjusted and adjusted ordinal logistic regression models predicting SRH for both groups. Unadjusted models demonstrated positive associations between literacy and SRH for both U.S.-born (UOR = 1.12, 95% CI = 1.10–1.14, p<.001) and immigrant adults (UOR = 1.09, 95% CI = 1.07–1.12, p<.001), and between numeracy and SRH for both U.S.-born (UOR = 1.09, 95% CI = 1.08–1.11, p<.001) and immigrant respondents (UOR = 1.08, 95% CI = 1.05–1.10, p<.001).

Adjusted models for literacy and SRH for U.S.-born and immigrant respondents are shown in Table 3. Controls for demographic and health characteristics (Models 1a and 1b) decreased the odds ratios for literacy among both U.S.-born and immigrant respondents, but literacy remained a significant predictor of SRH for both groups. The addition of human capital characteristics in Models 2a and 2b further attenuated the magnitude of the literacy odds ratio for U.S.-born respondents (AOR = 1.03, 95% CI = 1.01–1.05, p = .01), but the association between literacy and SRH remained significant. This suggests that for U.S.-born adults, human capital characteristics (formal education, employment, income, parental education) only partially explain the positive relationship between literacy and SRH. However, among immigrants, the introduction of human capital characteristics (Model 2b) decreased the odds ratio for literacy to an insignificant 1.00 (95% CI = 0.94–1.05, p = .88). This suggests that, among immigrants, the human capital characteristics that tend to be associated with assimilation—income, employment, education, speaking English well—explained the positive relationship between literacy and SRH.

**Table 3:** Adjusted Odds Ratios and 95% Confidence Intervals for Associations between Literacy and SRH by U.S.-Born versus Immigrant.

	U.S.-Born				Immigrants			
	Model 1a		Model 2a		Model 1b		Model 2b	
	AOR	p	AOR	p	AOR	p	AOR	p
Literacy	1.09 (1.07–1.11)	<.001	1.03 (1.01–1.05)	0.01	1.04 (1.00–1.08)	<.001	1.00 (0.94–1.05)	0.88
*Demographic Characteristics*								
Age								
24 or less (ref)	1		1		1		1	
25–34	0.73 (0.56–0.95)	0.02	0.67 (0.48–0.92)	0.01	0.36 (0.20–0.64)	<.001	0.25 (0.14–0.45)	<.001
35–44	0.53 (0.42–0.67)	<.001	0.50 (0.37–0.68)	<.001	0.28 (0.15–0.50)	<.001	0.18 (0.09–0.35)	<.001
45–54	0.48 (0.37–0.63)	<.001	0.49 (0.35–0.70)	<.001	0.22 (0.12–0.42)	<.001	0.14 (0.07–0.29)	<.001
55 or older	0.40 (0.31–0.52)	<.001	0.52 (0.38–0.71)	<.001	0.13 (0.06–0.26)	<.001	0.10 (0.04–0.21)	<.001
Female	0.92 (0.81–1.06)	0.26	0.95 (0.83–1.07)	0.26	0.81 (0.57–1.16)	0.25	0.80 (0.54–1.17)	0.25
Race/Ethnicity								
Non-Hispanic white (ref)	1		1		1		1	
Non-Hispanic black	0.88 (0.73–1.06)	0.19	0.87 (0.71–1.06)	0.17	1.03 (0.43–2.45)	0.95	0.70 (0.28–1.72)	0.43
Hispanic	0.91 (0.69–1.20)	0.52	1.07 (0.81–1.41)	0.65	0.66 (0.38–1.16)	0.15	0.80 (0.43–1.49)	0.47
Asian/Pacific Islander	0.88 (0.45–1.76)	0.73	0.84 (0.40–1.77)	0.64	0.69 (0.40–1.18)	0.17	0.69 (0.37–1.31)	0.26
Other race	0.75 (0.46–1.21)	0.23	0.89 (0.58–1.37)	0.59	2.42 (0.93–6.27)	0.07	2.47 (0.94–6.47)	0.07
Number of people living in household	1.01 (0.95–1.08)	0.71	1.04 (0.98–1.11)	0.21	0.92 (0.85–1.00)	0.05	0.96 (0.87–1.06)	0.43
Lives with a spouse or partner	1.27 (1.09–1.47)	0.002	1.09 (0.95–1.24)	0.22	1.32 (0.90–1.95)	0.16	1.31 (0.89–1.94)	0.17
Has children aged 12 or younger	1.03 (0.82–1.30)	0.81	0.99 (0.78–1.25)	0.93	1.23 (0.80–1.91)	0.35	1.22 (0.78–1.92)	0.39
Region								
West (ref)	1		1		1		1	
Northeast	1.19 (0.85–1.64)	0.31	1.13 (0.87–1.47)	0.36	1.35 (0.98–1.87)	0.07	1.42 (1.08–1.85)	0.01
Midwest	0.92 (0.67–1.26)	0.60	0.95 (0.73–1.22)	0.68	1.66 (1.04–2.65)	0.03	1.53 (1.01–2.32)	0.04
South	0.85 (0.64–1.12)	0.24	0.90 (0.72–1.14)	0.38	1.22 (0.83–1.81)	0.31	1.28 (0.91–1.81)	0.16
*Health Background*								
Has vision/hearing problems or diagnosed learning disability	0.49 (0.42–0.58)	<.001	0.63 (0.53–0.75)	<.001	0.33 (0.19–0.57)	<.001	0.42 (0.22–0.79)	0.01
Has health insurance	1.27 (1.06–1.52)	0.01	1.18 (0.98–1.43)	0.09	1.41 (0.99–2.02)	0.06	1.28 (0.83–1.96)	0.26
Received flu shot in past year	1.04 (0.90–1.20)	0.57	1.01 (0.87–1.17)	0.91	1.04 (0.75–1.43)	0.82	1.01 (0.72–1.41)	0.95
*Human Capital*								
Educational Attainment								
Did not complete high school (ref)			1					
Did not complete high school (ref)			1				1	
High school graduate/some college			1.07 (0.81–1.40)	0.64			0.93 (0.50–1.72)	0.82
Certificate from trade school or other			1.04 (0.71–1.54)	0.84			1.94 (0.79–4.80)	0.15
Associate degree			1.24 (0.88–1.75)	0.21			1.34 (0.62–2.91)	0.45
Bachelor’s degree			1.88 (1.34–2.64)	<.001			1.17 (0.47–2.93)	0.74
Master’s degree or higher			2.10 (1.42–3.10)	<.001			1.83 (0.88–3.84)	0.11
Employment Status								
Employed (ref)			1				1	
Unemployed			0.69 (0.46–1.03)	0.07			1.19 (0.63–2.24)	0.59
Pupil, student, apprentice, internship			1.01 (0.73–1.40)	0.94			0.91 (0.48–1.73)	0.78
Retired			0.73 (0.51–1.05)	0.09			0.56 (0.09–3.44)	0.53
Disabled			0.05 (0.03–0.07)	<.001			0.08 (0.02–0.33)	<.001
Homemaker or other			0.95 (0.67–1.36)	0.79			0.93 (0.55–1.58)	0.79
Income								
First quintile			0.76 (0.57–0.99)	0.04			0.67 (0.35–1.29)	0.23
Second quintile			0.87 (0.65–1.16)	0.35			0.95 (0.55–1.63)	0.85
Third quintile			0.97 (0.76–1.24)	0.79			0.77 (0.41–1.43)	0.40
Fourth quintile			0.92 (0.71–1.20)	0.55			1.92 (1.00–3.67)	0.05
Fifth quintile (ref)			1				1	
Income not reported			0.77 (0.62–0.96)	0.02			0.88 (0.52–1.50)	0.65
English proficiency level (higher score = better)			1.08 (1.01–1.15)	0.02			1.11 (1.02–1.20)	0.01
Mother’s Educational Attainment								
Did not complete high school (ref)			1				1	
Completed high school			1.30 (1.09–1.56)	0.004			0.97 (0.54–1.75)	0.92
Attended college or more			1.28 (1.01–1.62)	0.04			0.71 (0.44–1.13)	0.15
Father’s Educational Attainment								
Did not complete high school (ref)			1				1	
Completed high school			1.09 (0.94–1.27)	0.25			0.80 (0.48–1.32)	0.38
Attended college or more			1.44 (1.19–1.74)	<.001			1.02 (0.52–2.01)	0.94

N = 4,646 (U.S.-Born = 4,033; Immigrant = 613)

Two-tailed tests

AOR = Adjusted Odds Ratio

Adjusted models for numeracy and SRH for U.S.-born and immigrant respondents are shown in Table 4. Controls for demographic and health background characteristics (Models 1a) did little to reduce the magnitude of the odds ratio for U.S.-born (AOR = 1.07, 95% CI = 1.06–1.09, p<.001), but decreased the odds ratio for immigrants (Model 1b) from 1.08 in the unadjusted model to 1.03 (95% CI = 1.00–1.06, p = .02) in the adjusted model. This reduction was driven almost entirely by region of residence: Immigrants are more likely to live in the West than the Northeast or Midwest, where SRH is significantly better.

**Table 4:** Adjusted Odds Ratios and 95% Confidence Intervals for Associations between Numeracy and SRH by U.S.-Born versus Immigrant.

	U.S-Born				Immigrants			
	Model 1a		Model 2a		Model 1b		Model 2b	
	AOR	p	AOR	p	AOR	p	AOR	p
Numeracy	1.07 (1.06–1.09)	<.001	1.01 (0.99–1.03)	0.22	1.03 (1.00–1.06)	0.02	0.99 (0.96–1.03)	0.66
*Demographic Characteristics*								
Age								
24 or less (ref)	1		1		1		1	
25–34	0.71 (0.55–0.93)	0.01	0.66 (0.48–0.92)	0.01	0.36 (0.20–0.63)	<.001	0.25 (0.14–0.44)	<.001
35–44	0.52 (0.41–0.66)	<.001	0.50 (0.36–0.68)	<.001	0.27 (0.15–0.49)	<.001	0.18 (0.09–0.35)	<.001
45–54	0.47 (0.35–0.62)	<.001	0.49 (0.34–0.69)	<.001	0.22 (0.12–0.41)	<.001	0.14 (0.07–0.29)	<.001
55 or older	0.38 (0.29–0.50)	<.001	0.51 (0.37–0.70)	<.001	0.12 (0.06–0.25)	<.001	0.10 (0.04–0.21)	<.001
Female	1.02 (0.89–1.17)	0.79	0.96 (0.84–1.09)	0.52	0.84 (0.59–1.20)	0.35	0.79 (0.54–1.17)	0.24
Race/Ethnicity								
Non-Hispanic white (ref)	1		1		1		1	
Non-Hispanic black	0.94 (0.78–1.12)	0.49	0.85 (0.69–1.03)	0.10	1.03 (0.43–2.48)	0.94	0.68 (0.27–1.68)	0.40
Hispanic	0.88 (0.67–1.15)	0.34	1.04 (0.79–1.37)	0.78	0.65 (0.38–1.13)	0.13	0.78 (0.42–1.46)	0.44
Asian/Pacific Islander	0.88 (0.44–1.78)	0.73	0.83 (0.39–1.78)	0.64	0.68 (0.40–1.17)	0.16	0.69 (0.36–1.29)	0.24
Other race	0.76 (0.47–1.23)	0.27	0.89 (0.58–1.37)	0.59	2.48 (0.94–6.52)	0.07	2.46 (0.95–6.43)	0.07
Number of people living in household	1.01 (0.95–1.08)	0.68	1.04 (0.98–1.11)	0.23	0.92 (0.85–1.00)	0.06	0.96 (0.87–1.06)	0.43
Lives with a spouse or partner	1.25 (1.08–1.44)	0.003	1.09 (0.95–1.25)	0.21	1.29 (0.88–1.89)	0.19	1.32 (0.89–1.94)	0.17
Has children aged 12 or younger	1.02 (0.81–1.29)	0.87	0.90 (0.71–1.14)	0.37	1.23 (0.80–1.91)	0.35	1.22 (0.77–1.92)	0.39
Region								
West (ref)	1		1		1		1	
Northeast	1.17 (0.84–1.62)	0.34	1.12 (0.86–1.45)	0.41	1.38 (0.99–1.92)	0.05	1.41 (1.08–1.85)	0.01
Midwest	0.91 (0.67–1.24)	0.55	0.94 (0.73–1.21)	0.62	1.69 (1.06–2.69)	0.03	1.53 (1.01–2.31)	0.04
South	0.83 (0.63–1.09)	0.19	0.89 (0.71–1.12)	0.34	1.24 (0.85–1.83)	0.27	1.28 (0.91–1.80)	0.16
*Health Background*								
Has vision/hearing problems or diagnosed learning disability	0.48 (0.41–0.57)	<.001	0.62 (0.52–0.74)	<.001	0.33 (0.19–0.57)	<.001	0.42 (0.22–0.79)	0.01
Has health insurance	1.29 (1.08–1.54)	0.005	1.20 (0.99–1.45)	0.06	1.44 (1.02–2.03)	0.04	1.28 (0.84–1.95)	0.24
Received flu shot in past year	1.06 (0.92–1.21)	0.42	1.01 (0.87–1.17)	0.91	1.04 (0.75–1.44)	0.80	1.01 (0.72–1.41)	0.96
*Human Capital*								
Educational Attainment								
Did not complete high school (ref)			1				1	
High school graduate/some college			1.10 (0.84–1.43)	0.51			0.94 (0.51–1.73)	0.84
Certificate from trade school or other			1.07 (0.73–1.57)	0.73			1.97 (0.79–4.86)	0.14
Associate degree			1.30 (0.93–1.83)	0.13			1.36 (0.63–2.93)	0.43
Bachelor’s degree			1.99 (1.42–2.79)	<.001			1.20 (0.48–3.01)	0.70
Master’s degree or higher			2.25 (1.54–3.30)	<.001			1.90 (0.90–4.00)	0.09
Employment Status								
Employed (ref)			1				1	
Unemployed			0.68 (0.45–1.03)	0.07			1.19 (0.64–2.22)	0.58
Pupil, student, apprentice, internship			1.03 (0.75–1.43)	0.84			0.92 (0.49–1.73)	0.79
Retired			0.73 (0.52–1.04)	0.09			0.56 (0.09–3.49)	0.54
Disabled			0.04 (0.03–0.06)	<.001			0.08 (0.02–0.33)	0.00
Homemaker or other			0.96 (0.67–1.37)	0.81			0.93 (0.54–1.60)	0.80
Income								
First quintile			0.74 (0.56–0.97)	0.03			0.68 (0.36–1.29)	0.24
Second quintile			0.85 (0.64–1.13)	0.26			0.95 (0.56–1.60)	0.85
Third quintile			0.94 (0.74–1.20)	0.64			0.77 (0.42–1.42)	0.41
Fourth quintile			0.91 (0.71–1.18)	0.49			1.93 (1.01–3.70)	0.05
Fifth quintile (ref)			1				1	
Income not reported			0.76 (0.62–0.94)	0.01			0.88 (0.52–1.48)	0.63
English proficiency level (higher score = better)			1.09 (1.02–1.17)	0.01			1.11 (1.03–1.19)	0.01
Mother’s Educational Attainment								
Did not complete high school (ref)			1				1	
Completed high school			1.32 (1.10–1.58)	0.003			0.97 (0.54–1.76)	0.92
Attended college or more			1.31 (1.04–1.66)	0.02			0.71 (0.44–1.13)	0.15
Father’s Educational Attainment								
Did not complete high school (ref)			1				1	
Completed high school			1.10 (0.95–1.29)	0.21			0.80 (0.48–1.33)	0.40
Attended college or more			1.47 (1.21–1.77)	<.001			1.03 (0.53–2.02)	0.93

N = 4,646 (U.S.-Born = 4,033; Immigrant = 613)

Two-tailed tests

AOR = Adjusted Odds Ratio

The addition of human capital characteristics in Models 2a and 2b further decreased the odds ratios for numeracy for both U.S.-born and immigrant respondents to non-significance (U.S.-born AOR = 1.01, 95% CI = 0.99–1.03, p = .22; immigrant AOR = 0.99, 95% CI = 0.96–1.03, p = .66). Thus, as with literacy, human capital characteristics drove much of the relationship between numeracy and SRH for U.S.-born respondents.

Table 5 reports log odds from models that interacted immigrant status with literacy and numeracy to assess whether immigrants or U.S.-born respondents accrued more health benefits from literacy and numeracy proficiency. Because coefficients from interactions models cannot be interpreted in isolation from the main effects, we present log odds instead of odds ratios in these tables. Results demonstrated no significant interactions between immigrant status and proficiency in either literacy or numeracy on health. Both immigrants and U.S.-born respondents derive similar health rewards from literacy and numeracy proficiencies. Because interaction effects from these unadjusted models were not significant, we do not present results from adjusted models.

**Table 5:** Log Odds and Standard Errors from Ordinal Logistic Regression Models Interacting Immigrant Status x Literacy and Numeracy Proficiency to Predict SRH.

LITERACY	b	SE	p
Literacy	0.111	0.008	<.001
Immigrant	0.760	0.406	0.06
Literacy*Immigrant	-0.017	0.015	0.26
**NUMERACY**			
Numeracy	0.087	0.007	<.001
Immigrant	0.467	0.367	0.20
Numeracy*Immigrant	-0.011	0.014	0.43

N = 4,646

*Note*: two-tailed tests; SE = standard error

### Associations between Proficiency in Literacy and Numeracy and Self-Rated Health for Hispanic and Asian Immigrants

The remaining analyses were restricted to Hispanic and Asian immigrants. Their sample characteristics (Table 6) reveal that Asian immigrants had significantly higher average literacy and numeracy scores than Hispanic immigrants (p<.001). A significantly greater percentage of Asian immigrants reported very good health (36.3%) compared with Hispanic immigrants (24.6%, p = .01), and a significantly greater percentage of Hispanic immigrants reported fair health (18.9%) compared with Asian immigrants (6.4%, p<.001). In addition, a significantly greater percentage of Hispanic immigrants had a vision or hearing problem or learning disability (p = .015), but a significantly and substantially lower proportion had health insurance compared with Asian immigrants (p<.001), which likely reflects Hispanic immigrants’ higher likelihood of being undocumented [57]. Hispanic immigrants were also less likely to have received a flu shot in the past year (p = .031).

**Table 6:** Sample Characteristics for Hispanic and Asian Immigrants.

	Hispanic	Asian	t-value	p
Percentages or mean (standard deviation)	(N = 254)	(N = 166)		
Literacy Score^a^	210.38 (49.29)	264.64 (49.63)	-10.98	<.001
Numeracy Score^b^	192.27 (57.54)	257.68 (56.65)	-11.50	<.001
*Self-Rated Health*				
Excellent	19.9	25.6	-1.34	0.18
Very Good	24.6	36.3	-2.55	0.01
Good	34.3	28.2	1.33	0.16
Fair	18.9	6.4	4.03	<.001
Poor	2.3	3.5	-0.72	0.47
*Demographic Characteristics*				
Age				
24 or less	10.4	13.2	-0.86	0.39
25–34	27.8	27.6	0.03	0.98
35–44	25.0	27.6	-0.59	0.56
45–54	23.0	20.6	0.59	0.56
55 or older	13.8	10.9	0.88	0.38
Female	52.4	53.3	-0.18	0.86
Number of people living in household	4.16 (1.93)	3.47 (1.58)	4.24	<.001
Lives with a spouse or partner	67.9	67.9	-0.01	0.99
Has children aged 12 or younger	37.5	24.1	2.98	0.003
Region				
Northeast	12.8	32.9	-4.75	<.001
Midwest	3.6	11.6	-2.90	0.004
South	42.8	20.7	5.01	<.001
West	40.8	34.9	1.17	0.25
*Health Background*				
Has vision/hearing problems or diagnosed learning disability	22.6	13.5	2.44	0.02
Has health insurance	47.2	83.5	-8.51	<.001
Received flu shot in past year	38.8	49.7	-2.16	0.03
*Human Capital*				
Educational Attainment				
Did not complete high school	46.0	5.2	11.41	<.001
High school graduate/some college	35.0	25.6	2.08	0.04
Certificate from trade school or other	5.2	0.0	3.71	<.001
Associate degree	2.7	8.1	-2.28	0.02
Bachelor’s degree	7.0	29.9	-5.84	<.001
Master’s degree or higher	4.1	31.3	-7.13	<.001
Employment Status				
Employed	71.3	67.3	0.85	0.40
Unemployed	8.4	3.6	2.12	0.04
Pupil, student, apprentice, internship	4.5	11.6	-2.53	0.01
Retired	1.7	1.0	0.70	0.49
Disabled	2.5	3.4	-0.49	0.62
Homemaker or other	11.5	13.1	-0.49	0.63
Income				
First quintile	22.9	10.8	3.37	<.001
Second quintile	26.5	9.2	4.97	<.001
Third quintile	11.4	13.4	-0.58	0.57
Fourth quintile	5.2	11.2	-2.13	0.03
Fifth quintile	5.4	21.4	-4.58	<.001
Income not reported	28.6	34.1	-1.17	0.24
English proficiency level (higher score = better)	5.90 (4.04)	9.67 (2.84)	-11.99	<.001
Timing of Learning English				
Learned as first language	1.9	6.5	-2.12	0.04
Learned at age 15 or younger	39.3	80.9	-8.90	<.001
Learned at age 16 or older	58.5	12.5	10.52	<.001
Years in the US				
0–5 (ref)	9.0	19.0	-2.80	0.01
6–10	11.2	21.2	-2.65	0.01
11–15	23.6	22.4	0.29	0.77
More than 15 years	56.1	37.4	3.68	<.001
Mother’s Educational Attainment				
Did not complete high school	75.9	39.2	7.94	<.001
Completed high school	15.8	24.3	-2.13	0.03
Attended college or more	8.3	36.5	-6.83	<.001
Father’s Educational Attainment				
Did not complete high school	72.5	23.3	11.36	<.001
Completed high school	18.3	28.3	-2.36	0.02
Attended college or more	9.2	48.4	-9.12	<.001

^a^ Hispanic immigrants’ mean literacy score (210) corresponds to Level 1 (176–225), whereas Asian immigrants’ mean score (265) corresponds to Level 2 (226–275).

^b^ Hispanic immigrants’ mean numeracy score (192) corresponds to Level 1 (176–225), whereas Asian immigrants’ mean score (258) corresponds to Level 2 (226–275).

N = 420; weighted values

*Note*: Difference of means/proportions t-tests, p-values represent two-tailed tests

Hispanic immigrants had a higher average household size (p<.001) and were more likely than Asian immigrants to have young children (p = .003). Asian immigrants were more likely to live in the Northeast (p<.001) and Midwest (p = .004), and Hispanic immigrants were more likely to live in the South (p<.001). Over 80% of Hispanic immigrants live in just two regions (West and South), whereas Asian immigrants were more evenly dispersed across the four regions.

Substantial differences existed for human capital characteristics. Asian immigrants had significantly higher educational attainment than Hispanic immigrants. For example, 31.3% of Asian immigrants had a Master’s degree or higher, compared to only 4.1% of their Hispanic peers (p<.001). Hispanic immigrants were also more likely than Asian immigrants to be unemployed (p = .035) and less likely to be a student (p = .012). Asian immigrants reported significantly higher income, and importantly, there were no significant differences in the percentage of Hispanic versus Asian immigrants who did not report income from employment. There were also salient differences in English skills. Asian immigrants reported significantly higher English proficiency than Hispanic immigrants (p<.001) and were more likely to learn English as their first language (p = .035) or at age 15 or younger (p<.001). On average, Hispanic immigrants had been in the U.S. longer than Asian immigrants (e.g., 56% versus 37%, respectively, had resided in the U.S. for more than 15 years, p<.001). Finally, Asian immigrants reported much higher parental educational attainment. For instance, 36.5% of Asian mothers and 48.4% of Asian fathers attended college, compared to less than one-tenth of Hispanic mothers and fathers (p<.001). Overall, these results suggest that compared to their Asian peers, Hispanic immigrants are significantly disadvantaged in characteristics that are associated with health.

**Fig 3:**
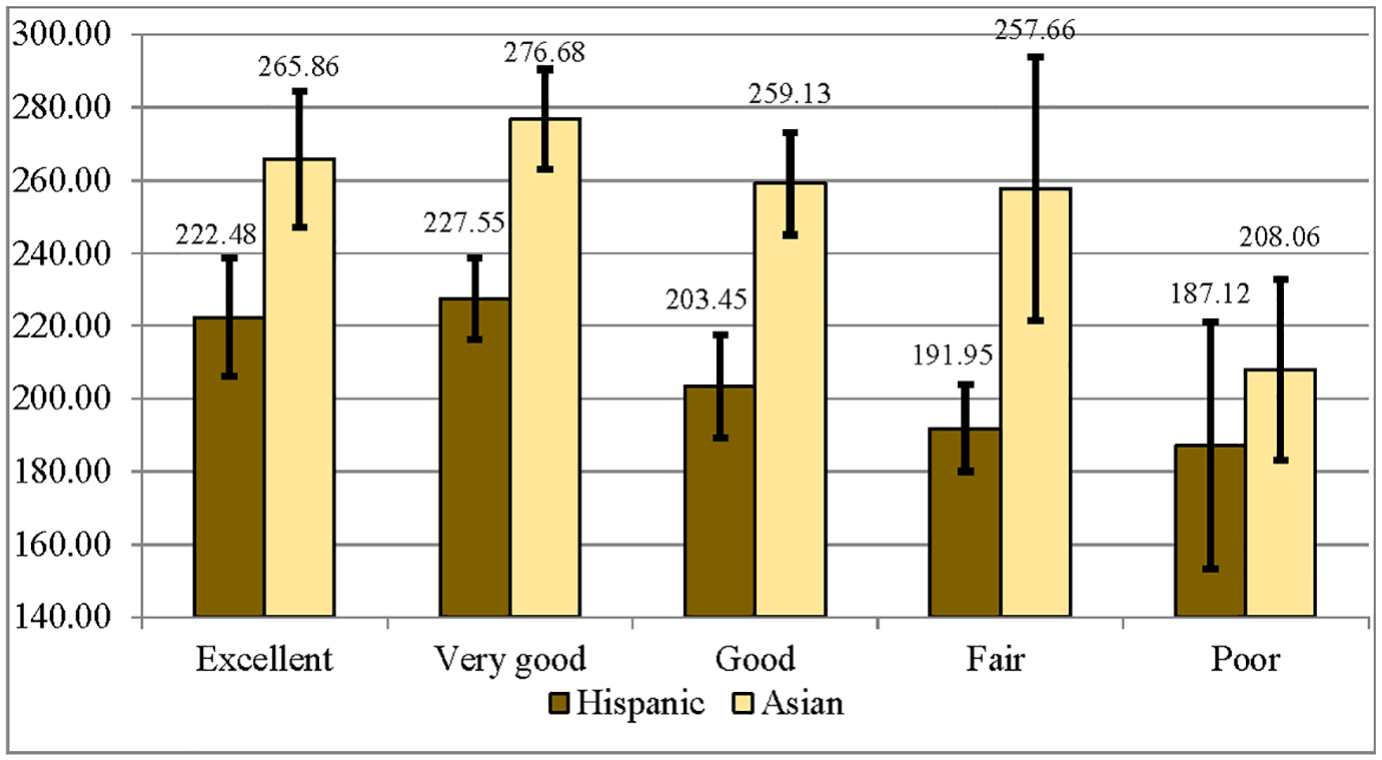
Average Literacy Scores by Self-Rated Health Category for Immigrant Hispanic and Asian Respondents (95% CIs).

Bivariate associations between literacy proficiency and SRH for Hispanic and Asian immigrants (Fig 3) demonstrate only minor associations between literacy and SRH. Among Hispanic immigrants, there were no significant differences in literacy scores across most SRH categories, as evidenced by confidence bars that mostly overlap). There were only two significant differences: Hispanic immigrants who reported *very good* health had a significantly higher average literacy score than those who reported *fair* health. Among Asian immigrants, those who reported *excellent* health or *very good* health had significantly higher average literacy scores than those who reported *poor* health.

**Fig 4:**
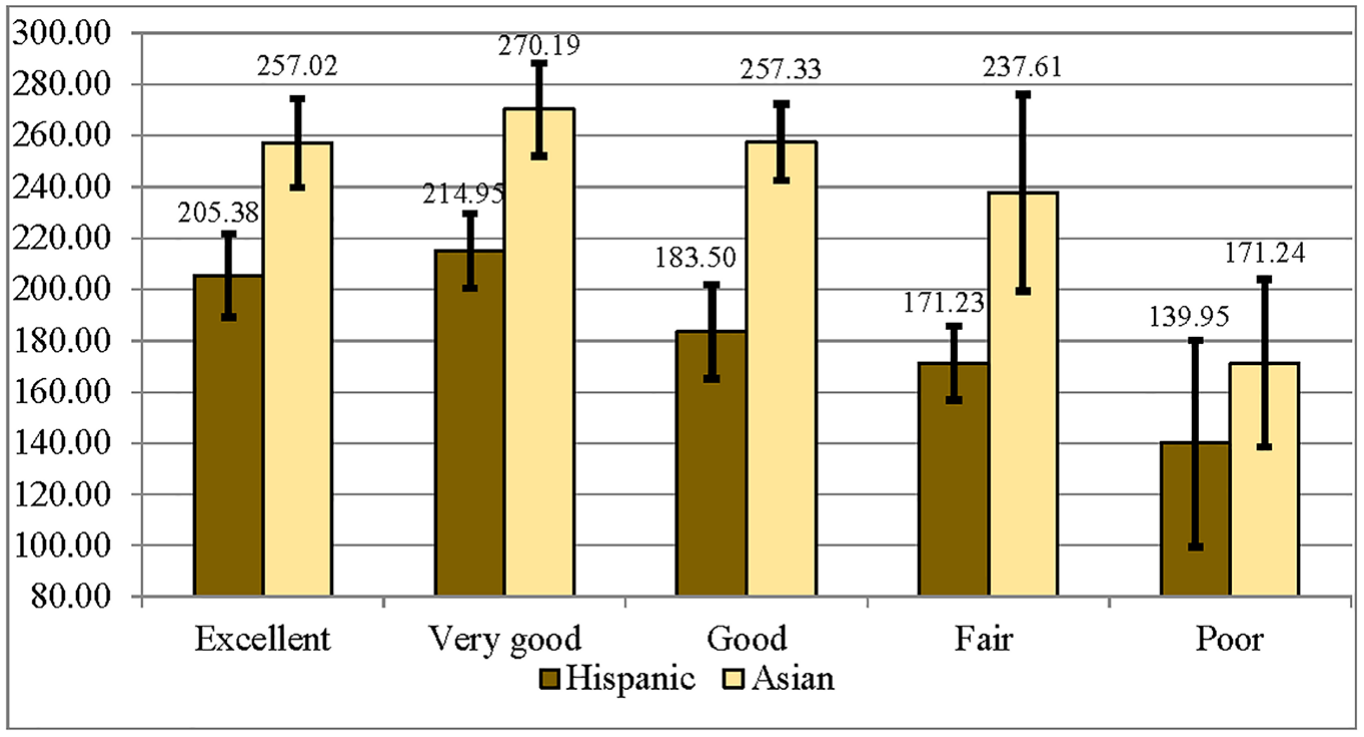
Average Numeracy Scores by Self-Rated Health Category for Immigrant Hispanic and Asian Respondents (95% CIs).

Results for bivariate association between numeracy and SRH are displayed in Fig 4. Hispanic immigrants who reported *excellent* or *very good* health had significantly higher numeracy scores than those who reported *fair* or *poor* health, and Asian immigrants who reported *poor* health had significantly lower average numeracy scores than those who reported *excellent*, *very good*, or *good* health.

Unadjusted ordinal logistic regression models predicting associations between literacy and SRH and numeracy and SRH for Hispanic immigrants revealed positive associations for both literacy and SRH (UOR = 1.11, 95% CI = 1.06–1.15, p<.001) and numeracy and SRH (UOR = 1.09, 95% CI = 1.05–1.14, p<.001). Among Asian immigrants, we found similarly positive associations between literacy and SRH (UOR = 1.07, 95% CI = 1.01–1.14, p = .02) and between numeracy and SRH (UOR = 1.05, 95% CI = 1.00–1.11, p = .046).

Adjusted models assessing associations between literacy and SRH for immigrant Hispanics and Asians are presented in Table 7. Due to small samples sizes for Hispanic (N = 254) and Asian (N = 166) immigrants, we did not have the statistical power to include all covariates in our regression models. Accordingly, we collapsed the categories of several control variables in our adjusted models. In addition to the controls included in the previous models, we also added timing of learning English and years in the U.S. as assimilation measures. Model 1a (Table 7) demonstrates that controlling for *demographic and health characteristics* reduced the odds ratio for literacy from 1.11 in the unadjusted model to 1.08 (95% CI = 1.03–1.14), but controlling for *human capital and assimilation characteristics* (Model 1b) eliminated the statistical significance for the association between literacy and SRH for Hispanics (AOR = 1.04, 95% CI = 0.95–1.13, p = 0.42). Among Asian immigrants, adding *demographic characteristics* (Model 1b) decreased the odds ratio from 1.07 in the unadjusted model to 1.06 (95% CI = 0.98–1.15, p = .12) in the adjusted model, eliminating the statistical significance. However, the reduction in p-value was due almost entirely to the increase in the standard error for literacy due to the addition of several covariates and the relatively small sample size, suggesting that the demographic characteristics themselves are not what led to the elimination of the significant association between literacy and SRH. Adding *human capital and assimilation characteristics* in Model 2b further reduced the odds ratio for literacy to 1.04 (95% CI = 0.92–1.17, p = 0.56).

**Table 7:** Adjusted Odds Ratios and 95% Confidence Intervals for Associations between Literacy and SRH by Immigrant Hispanic versus Immigrant Asian.

	Hispanic Immigrants (N = 254)				Asian Immigrants (N = 166)			
	Model 1a		Model 2a		Model 1b		Model 2b	
	AOR	p	AOR	p	AOR	p	AOR	p
Literacy	1.08 (1.03–1.14)	0.003	1.04 (0.95–1.13)	0.42	1.06 (0.98–1.15)	0.12	1.04 (0.92–1.17)	0.56
*Demographic Characteristics*								
Age								
under 55 (ref)								
55 or older	0.19 (0.09–0.42)	<.001	0.23 (0.07–0.70)	0.01	0.51 (0.17–1.56)	0.24	0.72 (0.31–1.68)	0.45
Female	0.57 (0.29–1.11)	0.10	0.62 (0.27–1.43)	0.26	0.87 (0.45–1.69)	0.68	1.01 (0.51–1.98)	0.98
Number of people living in household	0.94 (0.82–1.09)	0.43	0.97 (0.80–1.18)	0.79	1.07 (0.89–1.28)	0.46	1.22 (1.00–1.48)	0.05
Lives w/ spouse/partner	1.09 (0.60–1.98)	0.78	0.86 (0.39–1.89)	0.71	0.49 (0.25–0.96)	0.04	0.42 (0.21–0.86)	0.02
Has children aged 12 or younger	1.12 (0.55–2.29)	0.76	1.18 (0.45–3.10)	0.74	0.74 (0.32–1.74)	0.49	0.76 (0.36–1.61)	0.47
Region								
West (ref)								
Northeast	1.91 (0.97–3.78)	0.06	1.83 (0.77–4.35)	0.17	1.32 (1.21–1.45)	<.001	2.08 (0.90–4.80)	0.09
Midwest	1.05 (0.40–2.76)	0.92	1.07 (0.25–4.69)	0.93	1.49 (0.49–4.55)	0.49	2.25 (0.67–7.59)	0.19
South	1.76 (1.04–2.96)	0.03	2.64 (1.16–5.99)	0.02	0.74 (0.30–1.84)	0.52	1.05 (0.39–2.80)	0.93
*Health Background*								
Has vision/hearing problems or diagnosed learning disability	0.32 (0.14–0.73)	0.01	0.37 (0.14–0.99)	0.05	0.57 (0.13–2.40)	0.44	1.15 (0.26–5.04)	0.86
Has health insurance	1.44 (0.91–2.29)	0.12	1.37 (0.70–2.70)	0.36	1.60 (0.43–5.97)	0.49	2.01 (0.48–8.40)	0.34
Received flu shot in past year	1.56 (0.95–2.58)	0.08	1.57 (0.79–3.14)	0.20	1.12 (0.49–2.57)	0.79	0.83 (0.37–1.88)	0.66
*Human Capital*								
Educational Attainment								
Less than Master’s degree (ref)								
Master’s degree or higher			0.56 (0.24–1.28)	0.17			0.99 (0.44–2.24)	0.99
Employment Status								
Not employed (ref)								
Employed			0.50 (0.19–1.28)	0.15			2.35 (0.72–7.63)	0.16
Income								
Bottom 80th percentile (ref)								
Fifth quintile			2.70 (0.84–8.71)	0.10			0.65 (0.31–1.38)	0.27
Income not reported			1.02 (0.37–2.82)	0.95			0.98 (0.31–3.06)	0.97
English proficiency level (higher = better)			1.06 (0.94–1.19)	0.36			1.22 (0.92–1.60)	0.16
Timing of Learning English								
Learned as first language (ref)								
Learned at age 15 or younger			6.13 (0.18–212.4)	0.32			1.99 (0.69–5.68)	0.20
Learned at age 16 or older			4.75 (0.15–152.4)	0.38			0.92 (0.23–3.72)	0.91
Years in the US								
15 or fewer years (ref)								
More than 15 years			0.78 (0.40–1.50)	0.45			0.45 (0.24–0.82)	0.01
Mother’s Educational Attainment								
Did not complete high school/HS grad (ref)								
Attended college or more			0.87 (0.31–2.45)	0.80			0.51 (0.24–1.07)	0.08
Father’s Educational Attainment								
Did not complete high school/HS grad (ref)								
Attended college or more			1.12 (0.40–3.18)	0.83			1.95 (0.80–4.75)	0.14

*Note*: two-tailed tests

Adjusted models assessing associations between numeracy and SRH for immigrant Hispanics and Asians are presented in Table 8. Among Hispanic immigrants (Model 1a), the addition of *demographic characteristics* had little impact on the association between numeracy and SRH, but the addition of *human capital and assimilation characteristics* (Model 2a) decreased the odds ratio from 1.07 to a non-significant 1.02 (95% CI = 0.95–1.11, p = 0.58), suggesting that the relationship between numeracy and SRH for Hispanics may be largely driven by human capital and assimilation characteristics. Among Asian immigrants, the addition of *demographic characteristics* (Model 1b) did not change the odds ratio for numeracy (AOR = 1.05, 95% CI = 0.98–1.12, p = .18) compared to the unadjusted model, and the increase in the p-value was once again due to the loss of statistical power rather than the mediation effect of the control variables. The addition of *socioeconomic and assimilation characteristics* in Model 2b decreased the odds ratio for numeracy to 1.04 (95% CI = 0.94–1.15, p = .41). Overall, these results suggest positive relationships between literacy and numeracy and SRH for both Hispanic and Asian immigrants, with human capital and assimilation characteristics driving most of those associations.

**Table 8:** Adjusted Odds Ratios and 95% Confidence Intervals for Associations between Numeracy and SRH by Immigrant Hispanic versus Immigrant Asian.

	Hispanic Immigrants (N = 254)				Asian Immigrants (N = 166)			
	Model 1a		Model 2a		Model 1b		Model 2b	
	AOR	p	AOR	p	AOR	p	AOR	p
Numeracy	1.07 (1.02–1.12)	0.004	1.02 (0.95–1.11)	0.58	1.05 (0.98–1.12)	0.18	1.04 (0.94–1.15)	0.41
*Demographic Characteristics*								
Age								
under 55 (ref)								
55 or older	0.19 (0.09–0.40)	<.001	0.23 (0.07–0.69)	0.01	0.48 (0.16–1.41)	0.18	0.73 (0.31–1.71)	0.47
Female	0.62 (0.33–1.17)	0.14	0.63 (0.27–1.45)	0.28	0.98 (0.49–1.93)	0.95	1.07 (0.54–2.13)	0.84
Number of people living in household	0.96 (0.83–1.10)	0.53	0.98 (0.81–1.20)	0.87	1.07 (0.88–1.30)	0.50	1.24 (1.02–1.52)	0.03
Lives with a spouse or partner	1.03 (0.57–1.87)	0.92	0.84 (0.38–1.85)	0.66	0.47 (0.23–0.94)	0.03	0.39 (0.19–0.82)	0.01
Has children aged 12 or younger	1.12 (0.55–2.26)	0.76	1.17 (0.44–3.09)	0.75	0.76 (0.31–1.89)	0.56	0.73 (0.35–1.54)	0.41
Region								
West (ref)								
Northeast	2.11 (1.06–4.18)	0.03	1.90 (0.82–4.41)	0.13	1.34 (0.51–3.48)	0.55	2.09 (0.88–4.99)	0.10
Midwest	1.01 (0.39–2.61)	0.98	1.07 (0.24–4.74)	0.93	1.52 (0.47–4.93)	0.49	2.26 (0.63–8.07)	0.21
South	1.83 (1.10–3.03)	0.02	2.68 (1.17–6.09)	0.02	0.75 (0.30–1.88)	0.54	1.06 (0.39–2.88)	0.91
*Health Background*								
Has vision/hearing problems or diagnosed learning disability	0.33 (0.14–0.76)	0.01	0.37 (0.14–0.99)	0.05	0.56 (0.13–2.35)	0.43	1.23 (0.29–5.20)	0.77
Has health insurance	1.48 (0.93–2.35)	0.10	1.38 (0.69–2.73)	0.36	1.70 (0.45–6.40)	0.43	2.03 (0.50–8.17)	0.32
Received flu shot in past year	1.56 (0.94–2.58)	0.08	1.60 (0.80–3.22)	0.19	1.11 (0.48–2.58)	0.80	0.86 (0.38–1.96)	0.72
*Human Capital*								
Educational Attainment								
Less than Master’s degree (ref)								
Master’s degree or higher			0.57 (0.24–1.34)	0.20			0.96 (0.41–2.24)	0.92
Employment Status								
Not employed (ref)								
Employed			0.48 (0.19–1.22)	0.12			2.32 (0.72–7.46)	0.16
Income								
Bottom 80th percentile (ref)								
Fifth quintile			2.85 (0.90–9.01)	0.07			0.65 (0.31–1.37)	0.26
Income not reported			0.99 (0.36–2.68)	0.98			0.98 (0.32–3.06)	0.98
English proficiency level (higher score = better)							1.23 (0.93–1.62)	0.14
Timing of Learning English								
Learned as first language (ref)								
Learned at age 15 or younger			6.63 (0.20–219.8)	0.29			1.98 (0.75–5.20)	0.17
Learned at age 16 or older			4.93 (0.16–151.0)	0.36			0.86 (0.23–3.26)	0.82
Years in the US								
15 or fewer years (ref)								
More than 15 years			0.77 (0.39–1.52)	0.46			0.44 (0.24–0.80)	0.01
Mother’s Educational Attainment								
Did not complete high school/HS grad (ref)								
Attended college or more			0.87 (0.31–2.48)	0.80			0.52 (0.23–1.03)	0.06
Father’s Educational Attainment								
Did not complete high school/HS grad (ref)								
Attended college or more			1.14 (0.38–3.39)	0.81			1.90 (0.78–4.64)	0.16

*Note*: two-tailed tests

Results of interaction models (Table 9) showed no significant interactions between immigrant ethnic origin (i.e., Hispanic versus Asian) and proficiency in literacy or numeracy on SRH. We do not present results from adjusted models because they are repetitive. Ultimately, our results suggest that associations between literacy and numeracy proficiency and SRH are not stronger for one group of immigrants versus the other; both Hispanic and Asian immigrants attain similar SRH benefits from these skills.

**Table 9:** Log Odds and Standard Errors from Ordinal Logistic Regression Models Interacting Hispanic versus Asian x Proficiency in Literacy and Numeracy to Predict SRH.

LITERACY	Log odds	SE	p
Literacy	0.067	0.030	0.03
Hispanic	-1.032	0.841	0.22
Literacy*Hispanic	0.037	0.033	0.26
**NUMERACY**			
Numeracy	0.051	0.026	0.05
Hispanic	-1.062	0.779	0.17
Literacy*Hispanic	0.004	0.003	0.18

N = 420 (Hispanic = 254, Asian = 166)

*Note*: two-tailed tests; SE = standard error

## Discussion

Our results show that literacy and numeracy are both positively related to self-rated health, thus adding to the growing evidence that they are independent and significant social determinants of health. Importantly, these associations are almost entirely driven by human capital resources: educational attainment, parental education, employment, English proficiency, and low income.

Although cross-sectional data cannot be used to determine causality, we suggest two possible pathways through which literacy and numeracy proficiency may enhance health. First, literacy may enable people to obtain higher levels of education and better employment, which may lead to higher income—thus enabling people to acquire material resources needed for health (e.g., safe neighborhoods, high-quality health care)—and increased access to psychosocial resources and opportunities, including supportive social networks and a sense of control over their lives [20, 22–24] [28–31]. Second, educational attainment, English proficiency, and parents’ education (a strong correlate of children’s future education and income [58]) help cultivate literacy skills, which enable people to attain good employment and income, thereby helping them acquire health-promoting resources and the health benefits that come from being able to analyze health-related materials.

Immigrants’ lower scores on literacy and numeracy assessments administered in English are unsurprising. Thus, a policy implication is the need for basic skills instruction for immigrants, particularly those with the least formal schooling. However, despite immigrants’ low literacy and numeracy scores and disadvantaged socioeconomic position relative to their U.S.-born peers, they generally reported better health. This is consistent with prior large-scale literacy assessments [45] and research on the healthy immigrant effect [7–9]. Thus, the PIAAC data likely reflect healthy adults’ greater likelihood of migrating to the U.S.

Unlike research showing the “diminishing returns” of educational attainment for health among Blacks [3–5], we found no such pattern for literacy/numeracy proficiency and SRH. U.S.-born and immigrant respondents receive similarly positive health benefits from these proficiencies. This suggests that as a health promotion strategy, basic skills instruction would generate similar health benefits for both U.S.-born and immigrant adults.

Our comparisons of Asian versus Hispanic immigrants revealed that the latter were disadvantaged (relative to Asian immigrants) on almost all socioeconomic characteristics, including key determinants of health such as income, educational attainment, and access to health insurance. Moreover, they had much lower literacy and numeracy scores and worse SRH than Asian immigrants. However, for both groups, literacy and numeracy proficiency was positively related to SRH, and human capital and assimilation characteristics explained most of these associations, particularly for Hispanic immigrants. The finding of no statistical interaction is important because despite Hispanic immigrants’ lower literacy and numeracy scores and their disadvantaged socioeconomic position, our findings suggest that they receive equally positive health benefits from improving their literacy and numeracy abilities as Asian immigrants. As such, an important policy implication is to enhance literacy and numeracy—and thus indirectly promote health—for Hispanic immigrants, who have the fewest educational and socioeconomic resources and report worse health than Asian immigrants.

## Limitations

These results should be considered in light of some methodological limitations. First, the cross-sectional PIAAC data cannot be used to determine causal relationships between basic skills and health. Second, because the literacy and numeracy assessments were administered only in English, we could not assess how immigrants’ native language (L1) literacy and numeracy proficiency were related to their health. Similarly, the English-only assessment may have excluded immigrants with the least knowledge of written English, so our results may reflect the most English-proficient immigrants. Third, some of the PIAAC variables, such as parental education, are subject to recall bias. Fourth, the PIAAC (and other standardized basic skills instruments) measure *proficiency*, but they do not necessarily reflect an individual’s ability to critically evaluate or use health information [59] or the countless ways people use literacy and numeracy in their daily lives—what Reder calls practice engagement theory, whereby greater *use* of literacy improves proficiency over time [60]. Fifth, the PIAAC does not include a measure of documentation status. Because research suggests several barriers to good health for undocumented immigrants [61], controlling for documentation status may help to explain some of the differences in SRH and literacy and numeracy proficiency between Hispanic and Asian immigrants. Finally, there are some things the PIAAC data cannot tell us. For instance, the literacy scale chiefly measures reading comprehension rather than other dimensions of literacy such as writing, which may be crucial for managing and advocating for one’s health. Nor does the PIAAC does capture distributed or mediated literacy, that is, literacy and numeracy tasks that people accomplish with the help of knowledgeable others.

## Conclusion

Immigrants comprise a growing proportion of the U.S. population [14], and they come with variegated educational and health histories, socioeconomic resources, basic skill proficiencies, and ways of using literacy and numeracy. For immigrants and U.S.-born adults alike, health care is increasingly a literacy- and numeracy-demanding space [62], one that requires these abilities for full participation. As such, this research offers a timely analysis of how literacy and numeracy are related to health for foreign- versus U.S.-born adults and for Asian versus Hispanic immigrants—two large U.S. immigrant groups with divergent attributes and trajectories. Our study underscores the potential health benefits of providing adult basic education instruction for immigrants, especially those with the least formal schooling and fewest socioeconomic resources in their new country. Given the growing interest in adult education and health [15–19, 40, 63], we need more research on the best ways to integrate health-related topics into adult education and English language instruction for immigrants and U.S.-born adults who experience multiple forms of social exclusion.
